# Cardiac effects of 5F-Cumyl-PEGACLONE

**DOI:** 10.1007/s00414-023-03146-3

**Published:** 2024-01-12

**Authors:** Nicole Esdar, Evelyn Pawlik, Simon B. Eickhoff, Annika Raupach, Stefanie Ritz-Timme, Felix Mayer

**Affiliations:** 1 Institute of Legal Medicine, University Hospital Düsseldorf, 40225 Düsseldorf, Germany; 2 Departement of Trauma Surgery, Hand Surgery and Orthopedics, Petrus Hospital Wuppertal, 42283 Wuppertal, Germany; 3 Institute for Systems Neuroscience, University Hospital Düsseldorf, 40225 Düsseldorf, Germany; 4 Departement of Anaesthesiology, University Hospital Düsseldorf, 40225 Düsseldorf, Germany

**Keywords:** 5F-Cumyl-PEGACLONE, Synthetic cannabinoids, Cardiac adverse effects, Isolated perfused Langendorff heart

## Abstract

Synthetic cannabinoids become increasingly popular as a supposedly safe and legal alternative to cannabis. In order to circumvent the German New Psychoactive Substances Law, producers of so-called herbal mixtures rapidly design new substances with structural alterations that are not covered by the law. Acting as full agonists not only at the cannabinoid receptors 1 and 2, synthetic cannabinoids might have not only desired mental but also serious physical adverse effects. However, knowledge of adverse effects of specific substances is sparse and incomplete. This also accounts for 5F-Cumyl-PEGACLONE, a synthetic cannabinoid, which has been detected regularly in Germany in recent years. By using an animal model, the isolated perfused Langendorff heart, the study at hand aimed on finding out more about possible cardiovascular adverse effects of 5F-Cumyl-PEGACLONE. Hearts of male Wistar rats, which were excised postmortem, were exposed to two different concentrations of 5F-Cumyl-PEGACLONE: 13 hearts were exposed to 50 ng/ml and 12 hearts were exposed to 100 ng/ml. Thirteen control hearts were merely exposed to an additional amount of buffer solution. Functional parameters heart rate, minimal and maximum left ventricular pressure and coronary flow were documented at pre-defined time points during and after the administration of 5F-Cumyl-PEGACLONE/additional buffer solution. Electrocardiograms (ECGs) were documented throughout the experiments and evaluated afterwards. Kruskal–Wallis analysis was performed for each functional parameter as well as for the duration of the QRS complexes and the duration of RR intervals as derived from the ECGs. Furthermore, a multivariate analysis, comprising all functional and ECG parameters, was performed. Kruskal–Wallis analysis revealed only single significant *p*-values for QRS duration and minimum left ventricular pressure that did not pass a Bonferroni test. The results of the multivariate approach were also comparably homogeneous, but still the model correctly recognized hearts exposed to 100 ng/ml of 5F-Cumyl-PEGACLONE more often than hearts exposed to the low concentration of 5F-Cumyl-PEGACLONE or additional buffer solution. Evaluation of the ECGs presented single cases of ST depression and QT prolongation. Though certainly not unambiguous, these findings support the assumption that 5F-Cumyl-PEGACLONE can cause severe, if not lethal, cardiac adverse effects like arrhythmias or myocardial infarctions especially if it is consumed in combination with other drugs like alcohol or if the consumer suffers from pre-existing heart diseases.

## Introduction

After delta-9-tetrahydrocannabinol (THC) was discovered as a constituent of the cannabis plant with psychoactive effects, the first synthetic cannabinoids were synthesized in the 1960s. At the beginning of the millennium, they were increasingly sold commercially as “herbal mixtures” under brand names such as “Spice” or “Fake Pot” and established themselves from 2008 on as new designer drugs [1], which are usually smoked as a joint, from a water pipe [2] or an e-cigarette [3]. Through the unhindered purchase and the sellers’ marketing, the consumer is led to believe that the herbal mixtures are legal, safe and cheap alternatives to cannabis [4].

However, this presumption is misleading since synthetic cannabinoids, in contrast to the partial agonist THC, act as full agonists at the cannabinoid receptors 1 and 2 (CB1 and CB2) with a high activity potential. Interaction with these receptors, especially CB 1, is probably the main cause not only of mental effects, but also of physical adverse effects of synthetic cannabinoids which can affect the gastrointestinal, the cardiorespiratory and the nervous system [5]. The exact underling mechanisms, however, remain mostly unclear. Besides CB1 and CB2, synthetic cannabinoids are presumably also agonists at other receptors like the transient receptor potential vanilloid-type 1 (TRVP1) and the orphan G-protein-coupled receptor (GPR55) [5] as well as opioid and benzodiazepine receptors [1]. Their target receptors are found in many organs and tissues with CB1 being especially prevalent in the central nervous system [5]. Overall, a complex interaction of various mechanisms influencing the adrenergic system causing physical adverse effects has to be assumed, making the evaluation of an impact profile rather difficult. For example, different outcomes regarding cardiovascular effects of synthetic cannabinoids might be obtained in animal studies depending on the study setup with conscious or anesthetized animals [6].

In herbal mixtures, originally, a carrier substrate, e.g. plants like “Lion’s Tail” or “Indian Warrior” [7], was sprayed with or soaked in a liquid, in which the synthetic cannabinoids were previously dissolved and then dried. Today, producers of herbal mixtures typically simply add the synthetic cannabinoids as a powder to the dried plants. Both procedures lead to a considerable inhomogeneity of the active ingredients and the distribution within the mixtures [8, 9], though it seems to have decreased over the years [10]. Also, the brand name does not guarantee a constant composition of the ingredients within a product [11]. The range of variability makes it almost impossible for consumers to safely dose the synthetic cannabinoids contained in the herbal mixtures.

Initially, the German Narcotics Law (*BtMG*) did not provide a sufficient legal basis in the fight against the New Psychoactive Substances (NPS), because the inclusion of the individual substances was too slow. Although NPS have only few structural differences to substances already listed in the *BtMG*, these were already sufficient to circumvent penal provisions of the *BtMG* and thereby feign a supposed “legality.” Due to this problem, the New Psychoactive Substances Law (*NpSG*) came into force in November 2016 in Germany, which serves as an addition to the *BtMG*. Now, it is no longer the individual substance that is banned, but rather entire substance groups with defined structural characteristics. The substance group of synthetic cannabinoid receptor agonists (SCRAs) currently includes the core structures indoles, indazoles and benzimidazoles. However, the rapid proliferation of other new synthetic compositions that circumvent the defined structural features continues to challenge national legislation.

The SCRA 5F-Cumyl-PEGACLONE (5-(5-fluoropentyl)-2-(1-methyl-1-phenylethyl)-1H-pyrido[4, 3-b]indol-1-one) is a gamma-carboline-based synthetic cannabinoid, which represents one of such examples: in herbal mixtures, purchased in online shops during the EU project “SPICE Profiling,” the cannabinoid receptor agonist Cumyl-PEGACLONE (5-pentyl-2-(2-phenylpropan-2-yl)-2,5-di-hydro-1H-pyrido[4,3-b]indol-1-one) was detected in about 25% of all herbal blends, thereby leading the market [10]. After it was added to the *BtMG* in July 2018, its fluorinated analog 5F-Cumyl-PEGACLONE was increasingly found on the German market [4]. Both Cumyl-PEGACLONE and 5-F-Cumyl-PEGACLONE were shown to have a high CB 1 receptor activation potential [12, 13]. Considering its rising popularity, it is of particular interest which adverse effects can occur after the consumption of 5F-Cumyl-PEGACLONE. Even more so, as terminally fluorinated SCRAs are suggested to have a higher CB1 receptor activity [14]. Because of the possible dramatic results, adverse effects on the cardiovascular system are especially crucial. However, not only because of the above-mentioned uncertainties regarding the underlying mechanisms but also due to considerable differences between single SCRAs, there is only limited knowledge of the interaction between 5F-Cumyl-PEGACLONE and the cardiovascular system. To date, only some case reports exist that link cases of death to the consumption of Cumyl-PEGACLONE and/or 5F-Cumyl-PEGACLONE [15–18].

With the study at hand, we aimed on learning more about the direct effects 5F-Cumyl-PEGACLONE has on the heart. Working with an animal model, the isolated perfused Langendorff heart, various cardiac functional parameters were observed and documented when exposing rats’ hearts to 5F-Cumyl-PEGACLONE. The question concerning measurable effects of 5F-Cumyl-PEGACLONE on the functional parameters was tackled by choosing two different statistical approaches: a direct statistical comparison and a multivariate model.

## Material and methods

### The isolated perfused Langendorff heart

In general, the experimental setup was realized as described before [19, 20]: male Wistar rats aged 2 to 3 months with a weight between 250 g and 350 g were used for the experiment. They were sedated with an intraperitoneal injection of pentobarbital (80 mg/kg body weight; Narcoren, Merial, Germany) in a final volume of 2 ml NaCl (0.9%) containing 0.2 ml heparin (25,000 IU/5 ml; B. Braun, Melsungen, Germany), placed latero-proximal to the hind legs. After 5 min, sufficient depth of sedation was ensured by testing for the absence of reflexes. The rats were decapitated using a guillotine, followed by an immediate thoracotomy to remove the heart and connect it to the Langendorff system. The hearts were perfused with a previously prepared buffer solution at a constant system pressure of 80 mmHg. The buffer, a modified Krebs–Henseleit solution, was composed of the following components:

- 118 mM sodium chloride (VWR Chemicals Internationals GmbH, Belgium)
- 25 mM sodium hydrogen carbonate (Carl Roth GmbH + Co. KG, Germany)
- 11 mM D-glucose (Carl Roth GmbH + Co. KG, Germany)
- 4.7 mM potassium chloride (Merck Chemicals GmbH, Germany)
- 2.25 mM calcium chloride (Merck Chemicals GmbH, Germany)
- 1.2 mM magnesium sulfate hepta-hydrate (Carl Roth GmbH + Co. KG, Germany)
- 1.2 mM potassium dihydrogen phosphate (Merck Chemicals GmbH, Germany)
- 1 mM L-lactic acid sodium salt (AppliChem GmbH, Germany)
- 0.5 mM ethylenediaminetetraacetic acid (Carl Roth GmbH + Co. KG, Germany)

During the experiments, oxygen supply was ensured with a carbogen mixture of 95% O_2_ and 5% CO_2_. The maximal and the minimal left ventricular pressure (LVPmax and LVPmin) was measured by inserting a balloon into the left ventricle as a pressure gauge after removal of the left auricle. After a stabilization period of 15 min, electrocardiogram (ECG) electrodes were attached to the heart to measure cardiac electrical activity and the heart rate (HR). A total of three electrodes were placed, one at the aorta, one around the left coronary artery in close proximity to the location of the left auricle, and one at the cardiac apex. Coronary flow (CF) was measured by collecting and weighing the buffer solution that passed through the heart during a 1-min period.

Data were digitized using an analog to digital converter (PowerLab/8SP, ADInstruments Pty Ltd, Castle hill, Australia) and continuously recorded on a personal computer using LabChart Reader (ADInstruments) for Windows v5.0. The evaluation of the records regarding the functional parameters was done manually for the time points presented below.

### Study group

Based on the knowledge of concentrations of the predecessor drug Cumyl-PEGACLONE and of THC, two concentrations were determined that served as low and high doses of 5F-Cumyl-PEGACLONE. The low dose was defined as 50 ng/ml and the high dose as 100 ng/ml.

For the high dose of 100 ng/ml, 80 µl 5F-Cumyl-PEGACLONE were mixed with 7920 µl of the modified Krebs–Henseleit carrier solution. For the low dose of 50 ng/ml, 40 µl 5F-Cumyl-PEGACLONE were added to 7960 µl of modified Krebs–Henseleit carrier solution.

In total, the study group comprised 25 hearts. Of these, 13 hearts were exposed to 5 ml of the low dose solution of 5F-Cumyl-PEGACLONE (low dose group) and 12 hearts were exposed to 5 ml of the high dose solution of 5F-Cumyl-PEGACLONE (high dose group) by using a perfusor running at 30 ml/h over a time span of 10 min (drug phase).

**Fig. 1:**
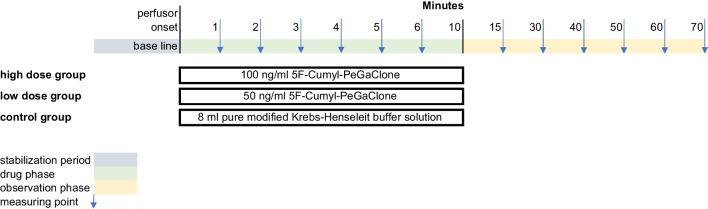
Study protocol of the experiments with the isolated perfused Langendorff heart

The recording of the relevant functional parameters (see above) started right before the onset of the perfusor, determining the individual base line values for each heart. After starting the perfusor, the designated parameters were measured at minutes 1, 2, 3, 4, 5, and 6 during the drug phase. Further measurements were performed at minutes 15, 30, 40, 50, 60, and 70 while hearts were, again, perfused only by the buffer solution (observation phase). Due to the manual measurement of CF, it was determined only before the onset of the perfusor and then at minutes 1 and 6 during the drug phase; during the observation phase, like the other parameters, it was measured at minutes 15, 30, 40, 50, 60, and 70. An overview of the study protocol is provided in Fig. 1.

### Control group

The control group comprised 13 hearts. Like in the study group, the hearts were connected to the Langendorff system and baseline values for each parameter were determined. During the drug phase, control hearts were only perfused with additional 5 ml of pure modified Krebs–Henseleit solution over a 10-min period. The measurements of the functional parameters were realized in the same way as in the study group.

### Electrocardiography

Besides a general assessment of all ECG graphs in terms of a “screening” for anomalies, the recorded data was manually evaluated by measuring the duration of the QRS complexes, as markers for the excitation propagation in the heart chambers, as well as the duration of RR intervals, as markers for the duration of an electrical heart action. They were measured at the same time points as all the other functional parameters, including the evaluation of a base line value right before the onset of the perfusor.

A general assessment of the QT interval was not possible because most ECGs did not show clear T waves. Therefore, this parameter was not included in the statistical analysis. Only two ECGs could be evaluated in this respect; the results are presented in a descriptive way.

### Statistics

One heart each from the high and the low dose group had to be excluded from the analysis because recording problems occurred during the experiments and thus, in retrospect, the documented values were not considered to be valid. Furthermore, one heart of the control group had to be excluded since the measured values presented massive and unexplainable deviations compared to the other hearts in this group. In summary, 11 hearts of the high dose group and 12 hearts each of the low dose group and of the control group, meaning 35 hearts in total, were included in the statistical analysis.

The acquired data were first analyzed by a univariate analysis procedure, the so-called Kruskal–Wallis analysis (one-way ANOVA of ranks), a statistical approach based on so-called rank sums which is suitable for the comparison of more than two groups. The null hypothesis assumes that there is no difference between the groups. Thus, in our model, the null hypothesis states that 5F-Cumyl-PEGACLONE has no measurable effect on the hearts’ function. In contrast, the H1 hypothesis states that 5F-Cumyl-PEGACLONE has a measurable effect on the hearts’ function.

The calculations were not based on the absolute measuring values, but on the deviation from the baseline as their reference value. For each measured parameter (LVP min, LVP max, CF, HR, QRS complex, RR interval), *p*-values were calculated at each measuring time point to detect a possible effect of the administered 5F-Cumyl-PEGACLONE. *p*-values < 0.05 were considered significant.

As a post hoc analysis, a Bonferroni test was performed on the results of the Kruskal–Wallis analysis.

In addition, a predictive model in terms of a multivariate analysis was applied. The model was trained on the data to learn differences in measurement patterns between the three groups, which could then be used to assign new, previously unseen cases (hearts) to the correct group (low dose group, high dose group, control group). The process was repeated several times so that different hearts could be left out and serve as the new, unseen case. The hit rate was put into a chart, with the *X*-axis showing the group which was predicted by the model and the *Y*-axis showing the true group in which the heart belonged.

## Results

### Kruskal–Wallis analysis

**Fig. 2:**
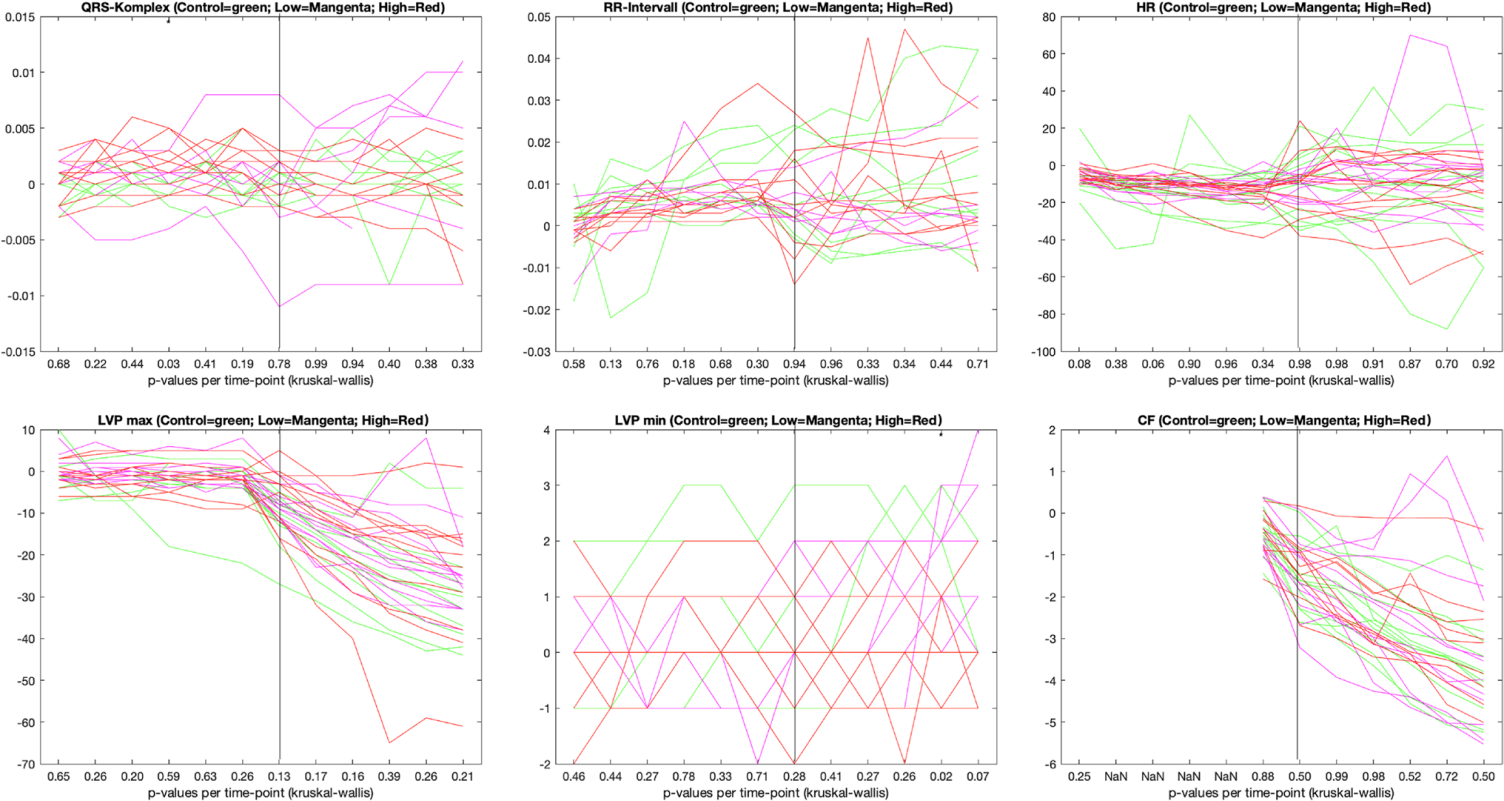
Results of the Kruskal–Wallis analysis for each measured parameter. The *Y*-axis presents the deviation from the baseline value. The *X*-axis shows *p*-values for each time point of measurement. The vertical straight line marks the time point of measurement 15 min after the start of the drug administration. Time points to the left represent measurements during the drug phase (minutes 1, 2, 3, 4, 5, and 6) and time points to the right further measurements during the observation phase (minutes 15, 30, 40, 50, 60, and 70). We refrained from a complete graph for cardiac flow during the drug phase, since it would have resulted in a straight line between the two measuring time points, falsely indicating a very homogeneous change. HR = heart rate; LVP max = maximum left ventricular pressure; LVP min = minimum left ventricular pressure; CF = cardiac flow

An overview of the results of the Kruskal–Wallis analysis is presented in Fig. 2.

### QRS complex

The calculated *p*-values per time point extend in a range from 0.03 at minute 4 to 0.99 at minute 30. The *p*-value of 0.03 was the only significant one, all other *p*-values were higher than 0.05. In general, *p*-values were slightly lower during the drug phase.

### RR interval

The range of calculated *p*-values per time point extends from 0.13 at minute 2 to 0.96 at minute 30. No significant *p*-values were found. *p*-values during the drug phase were comparably lower than during the observation phase.

### Heart rate

For the heart rate, the calculated *p*-values per time point extend in a range from 0.06 at minute 3 to 0.98 at minutes 15 and 30. No significant results were obtained. In general, *p*-values during the drug phase were slightly lower than during the observation phase.

### LVP max

The range of calculated *p*-values per time point for LVPmax extends from 0.13 (at minute 15) to 0.65 (at minute 1). No significant *p*-values were found. *p*-values during the observation phase were comparably lower than during the drug phase.

### LVP min

The range of *p*-values per time point for LVPmin extends from 0.02 to 0.78. The lowest value of 0.02 was calculated at minute 60. At minute 70, a second significant *p*-value with 0.07 was calculated. In general, *p*-values were lower during the observation phase than during the drug phase.

#### CF

The lowest *p*-value per time point for CF was calculated at minute 1 with 0.25; the highest *p*-value was calculated at minute 30 with 0.99. No significant *p*-values were obtained.

### Post hoc analysis

After performing the Bonferroni test, no significant results remained.

### Multivariate analysis

**Fig. 3:**
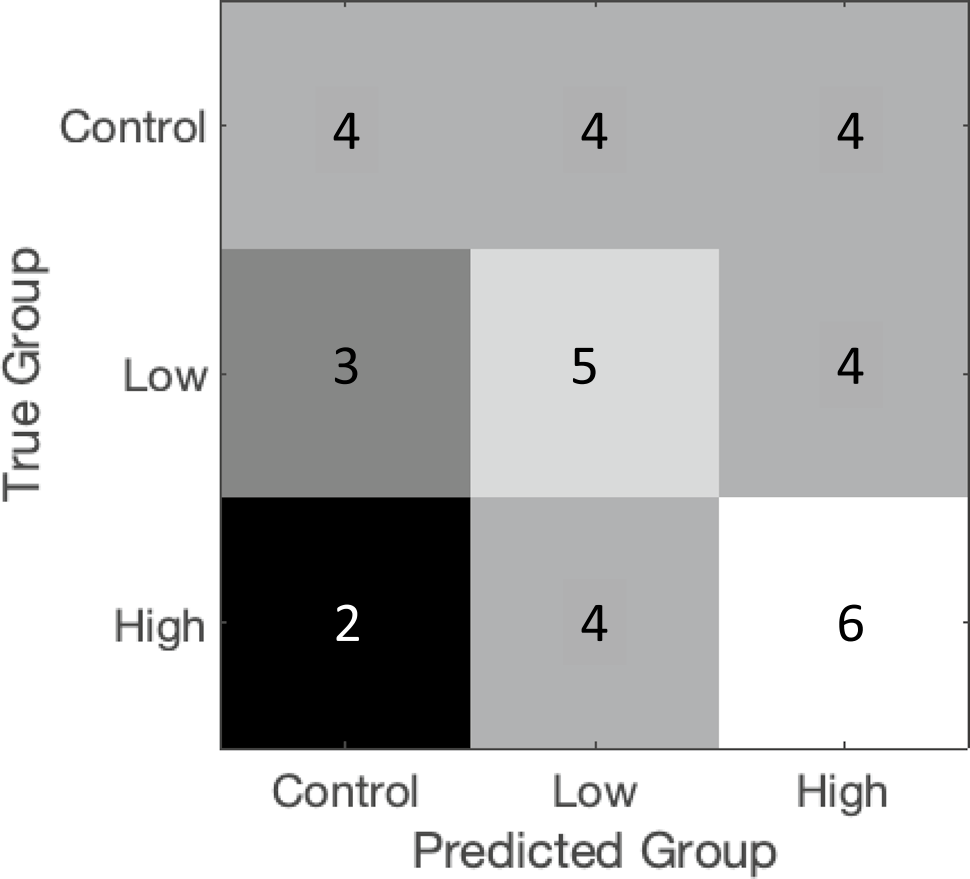
Results of the multivariate analysis approach. Numbers show, in which group measured values were sorted by a trained model compared to the true group they derived from. Control = control group; Low = low dose group; High = high dose group

A graphic depiction of the results is presented in Fig. 3.

The trained predictive model allocated 6 hearts of the high dose group correctly. However, 4 hearts exposed to the high dose of 5F-Cumyl-PEGACLONE were allocated to the low dose group and 2 hearts were allocated to the control group.

Five hearts of the low dose group were allocated correctly, whereas 4 hearts were allocated to the high-dose group and 3 hearts to the control group.

Results for the hearts of the control group were completely homogeneous with 4 hearts each being allocated to the control group, the low dose group, and the high dose group.

### Electrocardiography

In addition to the statistical evaluation, the graphically derived ECGs showed some pathological alterations in the LabChart plots.

### QRS complex

Two hearts presented ECG alterations that resembled QRS deformities indicating cardiac ischemia.

In the high dose group, one heart presented a clear reduction of the QRS amplitude in combination with an ST depression of about 0.4 mV from the ascending S from second 49 after the start of the perfusor. From minute 5, completely deformed QRS complexes with low voltage were seen for approximately 7 min. Subsequently, this complex pattern reappeared from minute 21 to minute 30 and then changed again to QRS complexes with visible ST depression.

**Fig. 4:**
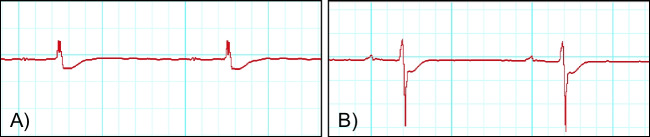
Examples of two hearts presenting ST depressions in the electrocardiogram graphs suggesting cardiac ischemia: **A** heart of the high dose group; **B** heart of the low dose group

In the low dose group, the ECG of one heart showed increasing ST depressions from the descending R-peak approximately 2 min after starting the perfusor with 5F-Cumyl-PEGACLONE. Subsequently, this was followed by increasing QRS deformity with concomitant low-voltage approximately 17 min after start of the perfusor. Examples of these ECG alterations are presented in Fig. 4.

### QT interval

The ECGs of two hearts, one heart of the high dose group and one heart of the control group, allowed the measurement of the QT duration. The heart of the control group presented a baseline QT duration of 0.057 ms. During the drug phase, only at minute 2, one longer QT interval of 0.058 ms was documented. All other measuring results ranged between 0.051 and 0.057 ms. The heart of the high dose group presented a baseline value of 0.053 ms. During the drug phase, the QT durations at minutes 1 to 4 were slightly longer with values ranging from 0.054 to 0.061 ms. Furthermore, one QT interval of 0.54 ms was detected during the observation phase at minute 16. The other measuring results ranged between 0.048 and 0.053 ms. At two time points, measurements were not possible because a clear T wave could not be determined.

## Discussion

It is well known that synthetic cannabinoids can have adverse effects on the cardiovascular system; however, detailed information about the specific impact of single substances are sparse and difficult to obtain. Cardiovascular symptoms of patients that consumed synthetic cannabinoids comprise not only tachycardia, being the most frequent symptom, as well as hypertension and chest pain, but also bradycardia and hypotension [1, 5, 21–24]. Also, arrhythmic effects of acute consumption of synthetic cannabinoids have been discussed [25] and the consumption of synthetic cannabinoids has been linked to severe cardiac events like myocardial infarctions [24, 26].

The model used in the study at hand, the isolated perfused Langendorff heart, can demonstrate direct effects of a substance on the heart and its function, depicted by alterations of various functional parameters. Regarding the results of the Kruskal–Wallis analysis of these parameters, it is obvious that only single measurements came up with significant differences between the study group and the control group which did not even pass the Bonferroni test. These significant differences concerned the QRS duration and LVP min.

Looking at the results of the Kruskal–Wallis analysis, the results of the multivariate approach do not come as a surprise. The trained model was only partly able to allocate single hearts correctly to one of the three groups, demonstrating that the differences between the groups are not distinct enough for such a classification. This becomes especially obvious when regarding the results of the control group being completely homogeneous. The results of the low dose group were only slightly better whereas the most correct allocations were obtained in the high dose group.

Still, it is questionable if the results of this study can serve as an “all-clear signal” for the consumption of 5F-Cumyl-PEGACLONE. Besides the possibility of a different picture in the entire organism as a “different setup”, a bolder interpretation of the results of the multivariate approach would mean that at least a tendency to recognizable alterations of cardiac functional parameters can be seen when hearts are exposed to a higher concentration of 5F-Cumyl-PEGACLONE. Individual risk profiles of consumers that might lead to different adverse effects also need to be taken into account. Only few case reports have been published that link the consumptions of 5F-Cumyl-PEGACLONE not only to severe, but lethal adverse effects. Giorgetti et al. [15] presented four cases in which adverse effects of 5F-Cumyl-PEGACLONE were discussed to be at least a contributing factor leading to death. In part, the cases went along with the consumption of various drugs being a typical risk factor for severe adverse effects. Tiemensma et al. [16], presenting a series of cases of fatalities linked to the consumption of Cumyl-PEGACLONE, claim that a concurrent use of alcohol might increase the risk for cardiac adverse effects as might underlying cardiovascular diseases. In contrast, a study from England did not find any deaths related to Cumyl-PEGACLONE in the years 2012–2019 [27], demonstrating the difficulty of evaluating the health risk of synthetic cannabinoids. In a German publication on deaths in the Munich area from 2014 to 2020 [18], 5F-Cumyl-PEGACLONE was mainly detected as part of polysubstance intoxications. Compared to the unfluorinated Cumyl-PEGACLONE, a higher toxicological significance score indicates a contribution to the fatality.

Some authors propose that a prolongation of the QT interval with the risk of cardiac arrythmias might be a serious adverse effect of SCRAs [25, 28–30]. The question, if this is the case for 5F-Cumyl-PEGACLONE cannot be answered with certainty by the results of our study. Only two hearts allowed the measurement of the QT interval with one heart of the high dose group showing prolongations during the drug phase; the second heart, on the other hand, belonging to the control group, presented no QT prolongation. Though certainly very sparse and not equivalent to a clinical ECG-evaluation, these findings might at least support the idea that 5F-Cumyl-PEGACLONE can have an impact of the electrical activity of the heart with the risk of serious arrhythmias. Furthermore, two hearts being exposed to 5F-Cumyl-PEGACLONE presented ECG-alterations, namely ST depressions, that imply cardiac ischemia and a risk for consumers to suffer from a myocardial infarction. Again, the risk for events like arrhythmias and myocardial infarctions is most likely higher if several drugs are consumed simultaneously and/or the consumer suffers from cardiovascular diseases.

## Limitations

For the study at hand, we worked with an animal model, the isolated perfused Langendorff heart. Insofar, the results do only reflect direct cardiac effects of 5F-Cumyl-PEGACLONE in an idealized, non-human setup. Furthermore, the evaluations of ECGs of the rats’ hearts under these conditions is certainly restricted compared to the evaluation of ECGs of human patients under clinical conditions. Also, the number of experiments is comparably low, judged from a statistical point of view. Still, we refrained from increasing the numbers due to ethical reasons. In addition to the low number of experiments, the interpretation is also limited by the low number of significant findings which did not pass the Bonferroni test. Therefore, conclusions can only be drawn from the tendencies of the multivariate approach. Unfortunately, the hearts were not preserved after the experiments so that histological examinations could not be performed.

## Conclusion

Kruskal–Wallis analysis came up with only single statistically significant alterations of cardiac functional parameters for rats’ hearts under the influence of 5F-Cumyl-PEGACLONE in the setup of the isolated perfused Langendorff heart; none of them passed the post hoc analysis. In a multivariate statistical approach, a trained model was only partly able to allocate cases/hearts correctly to the control group or one of the study groups. Still, the results of the study at hand imply that 5F-Cumyl-PEGACLONE might have an impact on the hearts’ function, also comprising the possibility of arrhythmias or myocardial infarctions. Especially when consumed simultaneously with other drugs or in cases of pre-existing cardiovascular diseases a risk for severe, possibly lethal adverse effects cannot be excluded.

## Data Availability

The data that support the findings of this study are available from the corresponding author, F. M., upon request.

## References

[1] Mills B, Yepes A, Nugent K (2015). Synthetic cannabinoids. Am J Med Sci.

[2] Tettey JNA, Crean C, Rodrigues J (2021). United Nations Office on Drugs and Crime: recommended methods for the identification and analysis of synthetic cannabinoid receptor agonists in seized materials. Forensic Sci Int Synerg.

[3] Lefever TW, Marusich JA, Thomas BF (2017). Vaping synthetic cannabinoids: a novel preclinical model of E-cigarette use in mice. Subst Abuse.

[4] Alam RM, Keating JJ (2020). Adding more “spice” to the pot: a review of the chemistry and pharmacology of newly emerging heterocyclic synthetic cannabinoid receptor agonists. Drug Test Anal.

[5] Le Boisselier R, Alexandre J, Lelong-Boulouard V, Debruyne D (2017). Focus on cannabinoids and synthetic cannabinoids. Clin Pharmacol Ther.

[6] Randall MD, Kendall DA, O’Sullivan S (2004). The complexities of the cardiovascular actions of cannabinoids. Br J Pharmacol.

[7] Lindigkeit R, Boehme A, Eiserloh I (2009). Spice: a never ending story?. Forensic Sci Int.

[8] Moosmann B, Angerer V, Auwärter V (2014). Inhomogeneities in herbal mixtures: a serious risk for consumers. Forensic Toxicol.

[9] Choi H, Heo S, Choe S (2013). Simultaneous analysis of synthetic cannabinoids in the materials seized during drug trafficking using GC-MS. Anal Bioanal Chem.

[10] Halter S, Mogler L, Auwarter V (2020). Quantification of herbal mixtures containing Cumyl-PEGACLONE-is inhomogeneity still an issue?. J Anal Toxicol.

[11] Shanks KG, Dahn T, Behonick G, Terrell A (2012). Analysis of first and second generation legal highs for synthetic cannabinoids and synthetic stimulants by ultra-performance liquid chromatography and time of flight mass spectrometry. J Anal Toxicol.

[12] Angerer V, Mogler L, Steitz JP (2018). Structural characterization and pharmacological evaluation of the new synthetic cannabinoid CUMYL-PEGACLONE. Drug Test Anal.

[13] Janssens L, Cannaert A, Connolly MJ, Liu H, Stove CP (2020). In vitro activity profiling of Cumyl-PEGACLONE variants at the CB(1) receptor: fluorination versus isomer exploration. Drug Test Anal.

[14] Banister SD, Stuart J, Kevin RC (2015). Effects of bioisosteric fluorine in synthetic cannabinoid designer drugs JWH-018, AM-2201, UR-144, XLR-11, PB-22, 5F-PB-22, APICA, and STS-135. ACS Chem Neurosci.

[15] Giorgetti A, Mogler L, Halter S (2019). Four cases of death involving the novel synthetic cannabinoid 5F-Cumyl-PEGACLONE. Forensic Toxicol.

[16] Tiemensma M, Rutherford JD, Scott T, Karch S (2021). Emergence of Cumyl-PEGACLONE-related fatalities in the Northern Territory of Australia. Forensic Sci Med Pathol.

[17] Schmidt B, Nigbur S, Kegler R, Büttner A, Rentsch D (2019). Letale Intoxikation unter Beteiligung des neuen synthetischen cannabinoids 5F-Cumyl-PeGaClone. Rechtsmedizin.

[18] Groth O, Roider G, Angerer V (2023). “Spice”-related deaths in and around Munich, Germany: a retrospective look at the role of synthetic cannabinoid receptor agonists in our post-mortem cases over a seven-year period (2014–2020). Int J Legal Med.

[19] Falk M, Huhn R, Behmenburg F, Ritz-Timme S, Mayer F (2018). Biomechanical stress in myocardial infarctions: can endothelin-1 and growth differentiation factor 15 serve as immunohistochemical markers?. Int J Legal Med.

[20] Gartz A, Pawlik E, Eckhardt J, Ritz-Timme S, Huhn R, Mayer F (2020). Effects of cocaine and levamisole (as adulterant) on the isolated perfused Langendorff heart. Int J Legal Med.

[21] Courts J, Maskill V, Gray A, Glue P (2016). Signs and symptoms associated with synthetic cannabinoid toxicity: systematic review. Australas Psychiatry.

[22] Muller HH, Kornhuber J, Sperling W (2016). The behavioral profile of spice and synthetic cannabinoids in humans. Brain Res Bull.

[23] Spaderna M, Addy PH, D’Souza DC (2013). Spicing things up: synthetic cannabinoids. Psychopharmacology (Berl).

[24] Alipour A, Patel PB, Shabbir Z, Gabrielson S (2019). Review of the many faces of synthetic cannabinoid toxicities. Ment Health Clin.

[25] Ozturk HM, Yetkin E, Ozturk S (2019). Synthetic cannabinoids and cardiac arrhythmia risk: review of the literature. Cardiovasc Toxicol.

[26] Pacher P, Steffens S, Hasko G, Schindler TH, Kunos G (2018). Cardiovascular effects of marijuana and synthetic cannabinoids: the good, the bad, and the ugly. Nat Rev Cardiol.

[27] Yoganathan P, Claridge H, Chester L, Englund A, Kalk NJ, Copeland CS (2022). Synthetic cannabinoid-related deaths in England, 2012–2019. Cannabis Cannabinoid Res.

[28] Von Der Haar J, Talebi S, Ghobadi F (2016). Synthetic cannabinoids and their effects on the cardiovascular system. J Emerg Med.

[29] Ozturk HM, Erdogan M, Alsancak Y (2018). Electrocardiographic alterations in patients consuming synthetic cannabinoids. J Psychopharmacol.

[30] Yun J, Yoon KS, Lee TH (2016). Synthetic cannabinoid, JWH-030, induces QT prolongation through hERG channel inhibition. Toxicol Res (Camb).

